# Manual of the Diseases of the Eye

**Published:** 1903-02

**Authors:** 


MANUAL OF THE DISEASES OF THE EYE.—For students and general practitioners; with 275 original illustrations, including 36 colored plates. By Chas. H. May, M. D., Chief of Clinic and Instructor in Ophthalmology, Eye Department, College of Physicians and Surgeons, Medical Department, Columbia University, New York. Second edition. Revised. Pages, 408. Price, $1.25. New York: Wm. Wood & Co.

This book is free from the common objections of too extensive detail and lengthy accounts of theories and rare conditions. The author is an experienced clinical teacher and understands how to stick to the subject for the purpose of good results. A didactic teacher should never write a book, because he generally gives what he has never seen and no one else, simply theory.

In this edition a chapter on ocular therapeutics has been added, besides a number of plates and colored illustrations. The work is an admirable one for students and general practitioners.

